# Authors' Reply to “Neuro-Ophthalmologic Complications of SARS-Cov-2 Infections and Vaccinations” [J Ophthalmic Vis Res 2024;19:140–141]

**DOI:** 10.18502/jovr.v21.13220

**Published:** 2026-02-04

**Authors:** Mehdi Tavakoli, Danielle R. Isen, Mohadeseh Feizi

**Affiliations:** ^1^George Washington University School of Medicine and Health System, Department of Ophthalmology, Washington, DC, USA; ^2^University of Alabama at Birmingham Heersink School of Medicine, Department of Ophthalmology and Visual Sciences, Birmingham, Alabama, USA; ^3^Ophthalmic Research Center, Research Institute for Ophthalmology and Vision Science, Shahid Beheshti University of Medical Sciences, Tehran, Iran

## Abstract

This letter responds to the letter titled “Neuro-Ophthalmologic Complications of SARS-Cov-2 Infections and Vaccinations,” published in J Ophthalmic Vis Res 2024;19:140–141 with DOI: https://doi.org/10.18502/jovr.v19i1.15451

##  Dear Editor,

We thank Dr. Finsterer and colleagues^[1]^ for their interest and scholarly commentary on our article entitled “Neuro-ophthalmic Manifestations of Coronavirus Disease 2019 and Its Vaccination: A Narrative Review.”^[1,2]^ The following provides our response to their concerns:

1. Our narrative review introduced and summarized updated information available about the neuro-ophthalmic manifestations of COVID-19 and its vaccination at the time of drafting. We aimed primarily to include patients who would present to a neuro-ophthalmologist in either an inpatient or outpatient setting. We aimed to present only the conditions for which these patients were referred to neuro-ophthalmology services, and not all of the ophthalmic conditions caused by COVID-19. Many of the conditions mentioned in the letter by Dr. Finsterer et al are usually referred to retina, uveitis, or external eye disease clinics. Among these conditions are Vogt-Koyanagi-Harada disease, multiple evanescent white dot syndrome (MEWDS), central retinal vein occlusion (CRVO), acute retinal necrosis (ARN), and Sjögren syndrome, which were categorized as omissions.

2. Headache is considered one of the most common conditions seen in a neuro-ophthalmology clinic.^[3]^ Not only is headache a leading symptom in some of the most common neuro-ophthalmic conditions, such as idiopathic intracranial hypertension and giant cell arteritis, but many of the primary headaches also originate or are perceived around the eyes. In addition, many other ocular conditions, such as dry eyes, uveitis, and glaucoma, may present with headaches rather than ocular pain. For these reasons, headache specialists often refer patients with headaches to neuro-ophthalmology for further evaluation. Moreover, headache and especially ocular pain are common in COVID-19 and can be an early sign of infection.^[4]^ Therefore, we think neuro-ophthalmologists must be aware of headache as a frequent manifestation of COVID-19.

3. Reversible cerebral vasoconstriction syndrome (RCVS) was mentioned as a disease not discussed in our review article. However, we did report two cases of posterior reversible encephalopathy syndrome (PRES), and many experts consider PRES to be on the same spectrum of disease as RCVS.^[5]^ Moreover, they commented that pituitary apoplexy was not included in our review, but it was addressed on page 116.

4. The emerging information on COVID-19 over the past three years has grown rapidly and evolved. While we reviewed and cited approximately 100 articles, it is possible that some other relevant articles may have been overlooked. Please note that there was also a gap between the time the manuscript was drafted and the final publication, and it is quite likely that we did not include articles added to the literature during this period.

We agree with Dr Finsterer et al^[1]^ that “neuro-ophthalmologic complications of SARS-CoV-2 infections and vaccinations are more diverse than is often assumed.” As we approach the end of the pandemic, future publications may provide a more comprehensive review of the ophthalmic, neurologic, and neuro-ophthalmic manifestations of COVID-19, as well as its vaccinations and treatments.
